# Diabetic phenotype and prognosis of patients with heart failure and preserved ejection fraction in a real life cohort

**DOI:** 10.1186/s12933-021-01242-5

**Published:** 2021-02-19

**Authors:** Sibille Lejeune, Clotilde Roy, Alisson Slimani, Agnès Pasquet, David Vancraeynest, Jean-Louis Vanoverschelde, Bernhard L. Gerber, Christophe Beauloye, Anne-Catherine Pouleur

**Affiliations:** Division of Cardiology, Department of Cardiovascular Diseases, Cliniques Universitaires St. Luc and Pôle de Recherche Cardiovasculaire (CARD), Institut de Recherche Expérimentale et Clinique (IREC), Cardiovascular Division, Université Catholique de Louvain, Avenue Hippocrate, 10, 1200 Brussels, Belgium

**Keywords:** Heart failure and preserved ejection fraction, Type 2 diabetes, HbA1C, Prognosis

## Abstract

**Background:**

Heart failure with preserved ejection fraction (HFpEF) is a heterogeneous syndrome, with several underlying etiologic and pathophysiologic factors. The presence of diabetes might identify an important phenotype, with implications for therapeutic strategies. While diabetes is associated with worse prognosis in HFpEF, the prognostic impact of glycemic control is yet unknown. Hence, we investigated phenotypic differences between diabetic and non-diabetic HFpEF patients (pts), and the prognostic impact of glycated hemoglobin (HbA1C).

**Methods:**

We prospectively enrolled 183 pts with HFpEF (78 ± 9 years, 38% men), including 70 (38%) diabetics (type 2 diabetes only). They underwent 2D echocardiography (n = 183), cardiac magnetic resonance (CMR) (n = 150), and were followed for a combined outcome of all-cause mortality and first HF hospitalization. The prognostic impact of diabetes and glycemic control were determined with Cox proportional hazard models, and illustrated by adjusted Kaplan Meier curves.

**Results:**

Diabetic HFpEF pts were younger (76 ± 9 vs 80 ± 8 years, p = 0.002), more obese (BMI 31 ± 6 vs 27 ± 6 kg/m^2^, p = 0.001) and suffered more frequently from sleep apnea (18% vs 7%, p = 0.032). Atrial fibrillation, however, was more frequent in non-diabetic pts (69% vs 53%, p = 0.028). Although no echocardiographic difference could be detected, CMR analysis revealed a trend towards higher LV mass (66 ± 18 vs 71 ± 14 g/m^2^, p = 0.07) and higher levels of fibrosis (53% vs 36% of patients had ECV by T1 mapping > 33%, p = 0.05) in diabetic patients.

Over 25 ± 12 months, 111 HFpEF pts (63%) reached the combined outcome (24 deaths and 87 HF hospitalizations). Diabetes was a significant predictor of mortality and hospitalization for heart failure (HR: 1.72 [1.1–2.6], p = 0.011, adjusted for age, BMI, NYHA class and renal function). In diabetic patients, lower levels of glycated hemoglobin (HbA1C < 7%) were associated with worse prognosis (HR: 2.07 [1.1–4.0], p = 0.028 adjusted for age, BMI, hemoglobin and NT-proBNP levels).

**Conclusion:**

Our study highlights phenotypic features characterizing diabetic patients with HFpEF. Notably, they are younger and more obese than their non-diabetic counterpart, but suffer less from atrial fibrillation. Although diabetes is a predictor of poor outcome in HFpEF, intensive glycemic control (HbA1C < 7%) in diabetic patients is associated with worse prognosis.

## Background

Heart failure with preserved ejection fraction (HFpEF) is increasingly being recognized as an umbrella term describing a heterogeneous group of clinical and pathophysiological phenotypes. HFpEF is a diagnostic challenge, especially since important features are mainly apparent on exercise and require dynamic testing [1]. Furthermore, the phenotypic heterogeneity among patients is a key reason for current lack of treatment improving outcome. Indeed most recent clinical trial using sacubitril-valsartan in HFpEF had disappointing results [2], although it could decrease the rate of hospitalisation in specific subgroups [3]. All eyes are now turned towards ongoing studies with sodium-glucose cotransporter-2 inhibitors (SGLT-2i) [4]. Type 2 diabetes (T2D) is one of the most frequent comorbidity associated with HFpEF (prevalence varying from 33 to 43%) [5], but there are still numerous uncertainties surrounding the mechanisms by which these two conditions interact. There is a need to understand the clinical characteristics of patients with HFpEF and diabetes in order to guide therapeutic decision making, highlight potential phenotype-specific targets, and aid in the development of risk stratification tools. Sub studies of large clinical trials (RELAX-HF [6], I-PRESERVE [7], CHARM [8] and TOPCAT [9]) comparing diabetic and nondiabetic patients showed that HFpEF patients with T2D were younger, more obese, displayed greater structural echocardiographic abnormalities (higher left ventricular mass) and had a worse prognosis than patients without T2D. Those studies were clinical trials with restrictive inclusion criteria and might not reflect HFpEF patients encountered in daily practice. The same differences in clinical characteristics were found in a large American registry (GWTG-HF registry) [10] but imaging parameters were not available for analyzes. Previous studies [11, 12] showed a U-shaped association between HbA1C and prognosis in heart failure patients. Those studies either were conducted among patients with HFrEF alone, or did not make a distinction between patients according to ejection fraction. Glycemic variability was found to be associated with diastolic dysfunction and with poor outcome in HFpEF [13, 14], but data remain limited. Accordingly, we aimed to investigate phenotypic differences between diabetic and nondiabetic patients with HFpEF in a prospective, real life cohort. The prognostic impact of glycemic control assessed by HbA1C was also evaluated in this population.

## Methods

### Study population

Patients with HFpEF encountered in our division of cardiology between December 2015 and June 2017 (in hospital and at ambulatory visits) were prospectively screened for inclusion in the study. Inclusion and exclusion criteria were reported in previous publications [15]. Briefly, the following criteria had to be fulfilled: New York Heart Association (NYHA) functional class ≥ II, typical signs of HF, NT-proBNP > 350 pg/ml and/or an hospitalization for HF in the previous 12 months, left ventricular ejection fraction ≥ 50%, and relevant structural heart disease (left ventricular (LV) hypertrophy/left atrial (LA) enlargement) and/or diastolic dysfunction by echocardiography [16]. The exclusion criteria were: history of reduced ejection fraction (LVEF < 50%), severe valvular disease, infiltrative or hypertrophic cardiomyopathy, acute coronary syndrome in the previous 30 days, severe chronic obstructive pulmonary disease, congenital heart disease, pericardial disease, atrial fibrillation (AF) with a ventricular response > 140 bpm, and severe anemia (hemoglobin < 8 g/dl). A total of 183 patients satisfied the inclusion criteria. Patients underwent blood sampling and complete transthoracic echocardiography and cardiac magnetic resonance (CMR) in the absence of following contra-indications: pacemaker, claustrophobia or estimated glomerular filtration rate (eGFR) < 30 mL/min/1.73 m^2^ (N = 151). The local ethics committee approved the study, and all patients gave written informed consent before study enrolment (Clinical trial NCT03197350). The investigation conforms to the principles outlined in Declaration of Helsinki.

### Clinical data

Patients were interrogated about symptoms, medical history and treatment and were thoroughly examined. Other information, including diagnosis and treatment of diabetes were retrieved from medical files and from review of hospital records.

### Echocardiography

Standardized complete transthoracic echocardiography (TTE) exams were acquired according to established guidelines using iE33 ultrasound systems (Philips Medical Systems, Andover, Massachusetts) equipped with a 3.5/1.75-MHz phased-array transducer and stored on a XCELERA 2.1 PACS server (Philips Medical Systems, Andover, Massachusetts).

### Cardiac magnetic resonance

CMR was performed using a 3 T system (Ingenia, Philips Medical Systems, Best, The Netherlands). The different sequences have been previously described [17]. Pre- and post-contrast MOLLI images were processed using the open-source software MRmap v1.4 under IDL. Pre- and post-myocardial T1 times were measured in six regions of interest in the myocardium (anterior, anterolateral, inferolateral, inferior, inferoseptal, anteroseptal). We calculated the average T1 time of the six different regions of interest. Areas of ischemic focal fibrosis identified by late gadolinium enhancement (LGE) were excluded from the analysis. Extracellular volume (ECV) was then computed according to the formula [18]. A cut off of ECV > 33% was used to define significant diffuse myocardial fibrosis [17].

### Follow up

Patients were prospectively followed by ambulatory visits and phone calls at 6-months intervals. Clinical and survival status was obtained by follow up visits and by phone contact with the patients, their relatives, or their physician if necessary. The primary endpoint was a composite of all-cause mortality or hospitalization for HF, whichever came first. Hospitalization was defined as patients diagnosed with heart failure and requiring intravenous diuretics, either treated in the emergency room or admitted to the hospital.

### Statistical analysis

Statistical analyses were performed using SPSS version 25 (SPSS Corp., Somers, New York). All tests were 2-sided and p-value < 0.05 was considered statistically significant. Continuous variables were expressed as mean ± 1 standard deviation (SD) and categorical variables as count and proportion. Differences of characteristics between groups were examined using independent sample t-test or Chi square test when appropriate. Uni- and multivariate Cox regression analyzes were used to determine the prognostic impact of diabetes and HbA1C. Diabetic patients enrolled in the study who completed the follow up (67/70, 96%) and with at least one HbA1c measurement in the three months previous to inclusion were used for analyzes about the prognostic impact of glycemic control (62/70, 89%) (Additional file 1). Adjusted Kaplan Meier curves were used to illustrate event-free survival of HFpEF patients. The log-rank test was used to compare survival among different groups.

## Results

### *Characteristics and outcome of diabetic versus nondiabetic HFpEF patients (Table *1***)***

**Table 1:** Clinical, echocardiographic and CMR characteristics of diabetic versus nondiabetic HFpEF patients

	Nondiabetic N = 113 (62%)	Diabetic N = 70 (38%)	p-value
Age (years)	80 ± 8	76 ± 9	0.002
Female (n, %)	71 (63%)	42 (60%)	0.70
Body mass index (kg/m^2^)	27 ± 6	31 ± 6	0.001
NYHA III–IV (n, %)	60 (53%)	27 (39%)	0.056
Hospitalized for HF at inclusion (n,%)	73 (65%)	43 (61%)	0.53
Atrial fibrillation (n, %)			
History	78 (69%)	37 (53%)	0.028
At inclusion	57 (50%)	26 (37%)	0.079
Ischemic cardiomyopathy (n, %)	27 (24%)	33 (47%)	0.001
Smoking (n, %)	50 (45%)	27 (39%)	0.42
Hypertension (n, %)	105 (93%)	67 (97%)	0.23
Hypercholesterolemia (n, %)	66 (59%)	49 (70%)	0.13
Sleep apneas (n, %)	8 (7%)	12 (18%)	0.032
COPD (n, %)	12 (11%)	7 (10%)	0.88
Medication			
Loopdiuretics (n, %)	73 (65%)	51 (73%)	0.25
MRA (n, %)	19 (17%)	13 (19%)	0.76
Beta blockers (n, %)	77 (68%)	41 (59%)	0.19
ACE inhibitors/ARB (n, %)	78 (69%)	46 (66%)	0.64
Biology			
eGFR (ml/min/1.73m^2^)	58 ± 22	50 ± 24	0.026
Hemoglobin (g/dL)	12 ± 2	11 ± 2	0.041
NT-proBNP (pg/mL)	1937 [1040–3775]	1745 [955–3710]	0.56
Troponin (pg/mL)	22 [13–37]	31 [17–42]	0.034
Echocardiography			
Indexed LA volume (mL/m^2^)	46 ± 19	45 ± 16	0.67
LV ejection fraction (%)	62 ± 7	61 ± 8	0.35
E wave velocity (mm/s)	91 ± 32	97 ± 26	0.23
Septal E/e’	19 ± 9	20 ± 7	0.17
TAPSE (mm)	19 ± 5	18 ± 5	0.40
eSPAP (mmHg)	43 ± 11	45 ± 15	0.27
CMR	N = 94	N = 57	
CMR indexed LA Volume (mL/m^2^)	70 ± 31	62 ± 25	0.12
CMR indexed LV EDV (mL/m^2^)	72 ± 18	74 ± 17	0.37
CMR LV ejection fraction (%)	62 ± 8	62 ± 9	0.62
CMR indexed LV mass (g/m^2^)	66 ± 18	71 ± 14	0.07
CMR RV ejection fraction (%)	56 ± 8	58 ± 8	0.41
CMR indexed RV EDV (mL/m^2^)	79 ± 25	83 ± 27	0.36
ECV > 33%	34 (36%)	28 (53%)	0.05

Continuous variables are expressed as mean ± 1 standard deviation (SD) and categorical variables as count and proportion. P-values are derived from independent sample t-test or Chi square test when appropriate

NYHA: New York Heart Association; COPD: chronic obstructive pulmonary disease; GFR: glomerular filtration rate estimated by CKD-epi; MRA: mineralocorticoid receptor antagonist; ACE: angiotensin-converting enzyme; ARB: angiotensin II receptor blockers LV: left ventricle; LA: left atrium; TAPSE: tricuspid annular plane systolic excursion; eSPAP: estimated systolic pulmonary artery pressures; CMR: cardiac magnetic resonance; EDV: end diastolic volume; RV: right ventricle; ECV: extracellular volume estimated by T1 mapping

**Fig. 1:**
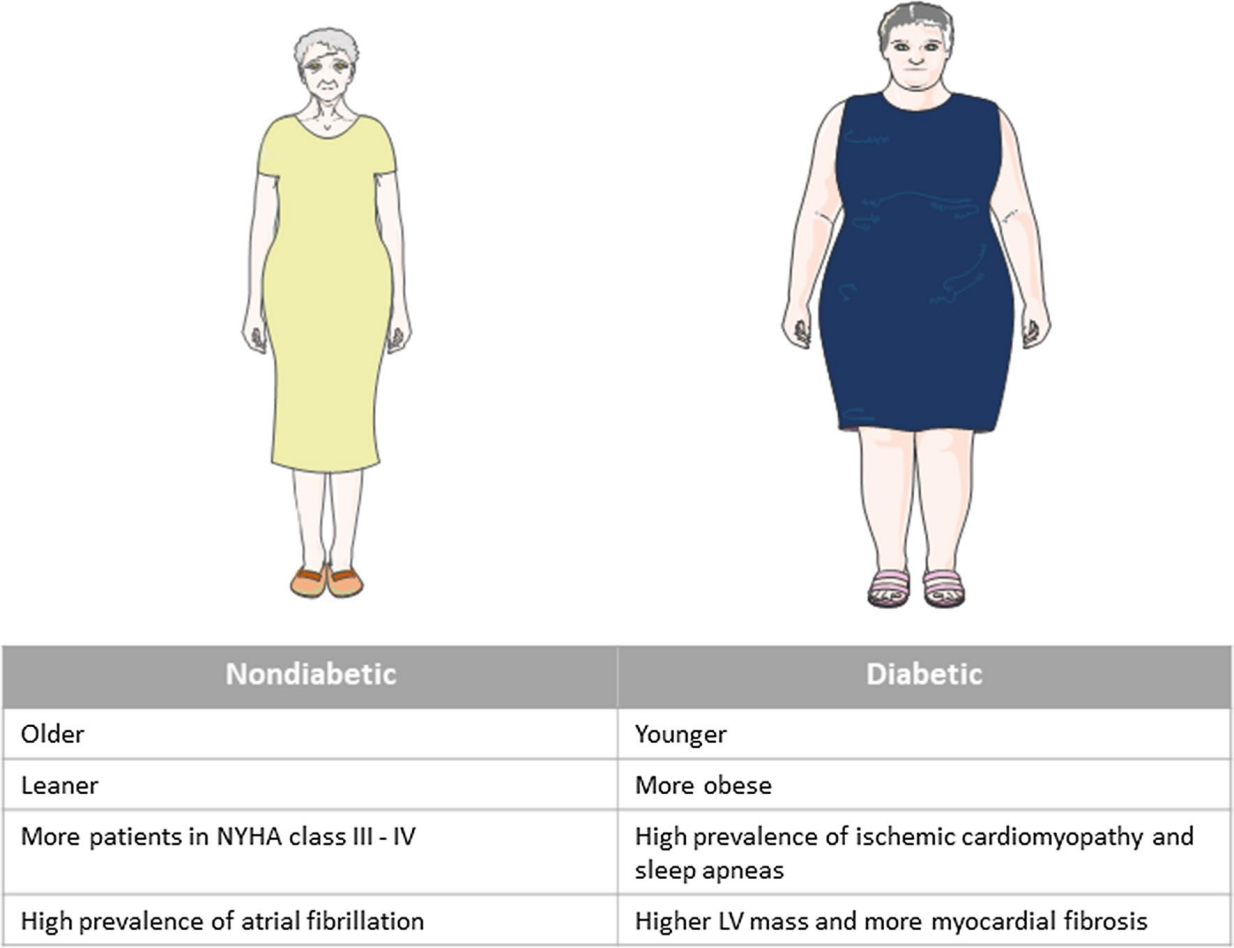
Characteristics of diabetic versus nondiabetic HFpEF patients

The total population was constituted of 183 HFpEF patients (78 ± 9 years, 62% women), including 70 (38%) diabetics. Diabetic HFpEF patients were younger (76 ± 9 vs 80 ± 8 years, p = 0.002) and more obese (body mass index (BMI) 31 ± 6 vs 27 ± 6 kg/m^2^, p = 0.001). They suffered more frequently from chronic coronary artery disease (47% vs 24%, p = 0.001) and obstructive sleep apnea (18% vs 7%, p = 0.032). Atrial fibrillation, however, was more frequent in nondiabetic patients (69% vs 53%, p = 0.028). Although no echocardiographic difference could be detected between the two groups, CMR analysis revealed a trend towards higher LV mass in the diabetic population (66 ± 18 vs 71 ± 14 g/m^2^, p = 0.07). Interestingly, more diabetic patients (53% vs 36%, p = 0.05) had high levels of myocardial fibrosis (defined as ECV by T1 mapping > 33%) [17]. The main differences between diabetic and nondiabetic patients are summarized in Fig. 1.

**Fig. 2:**
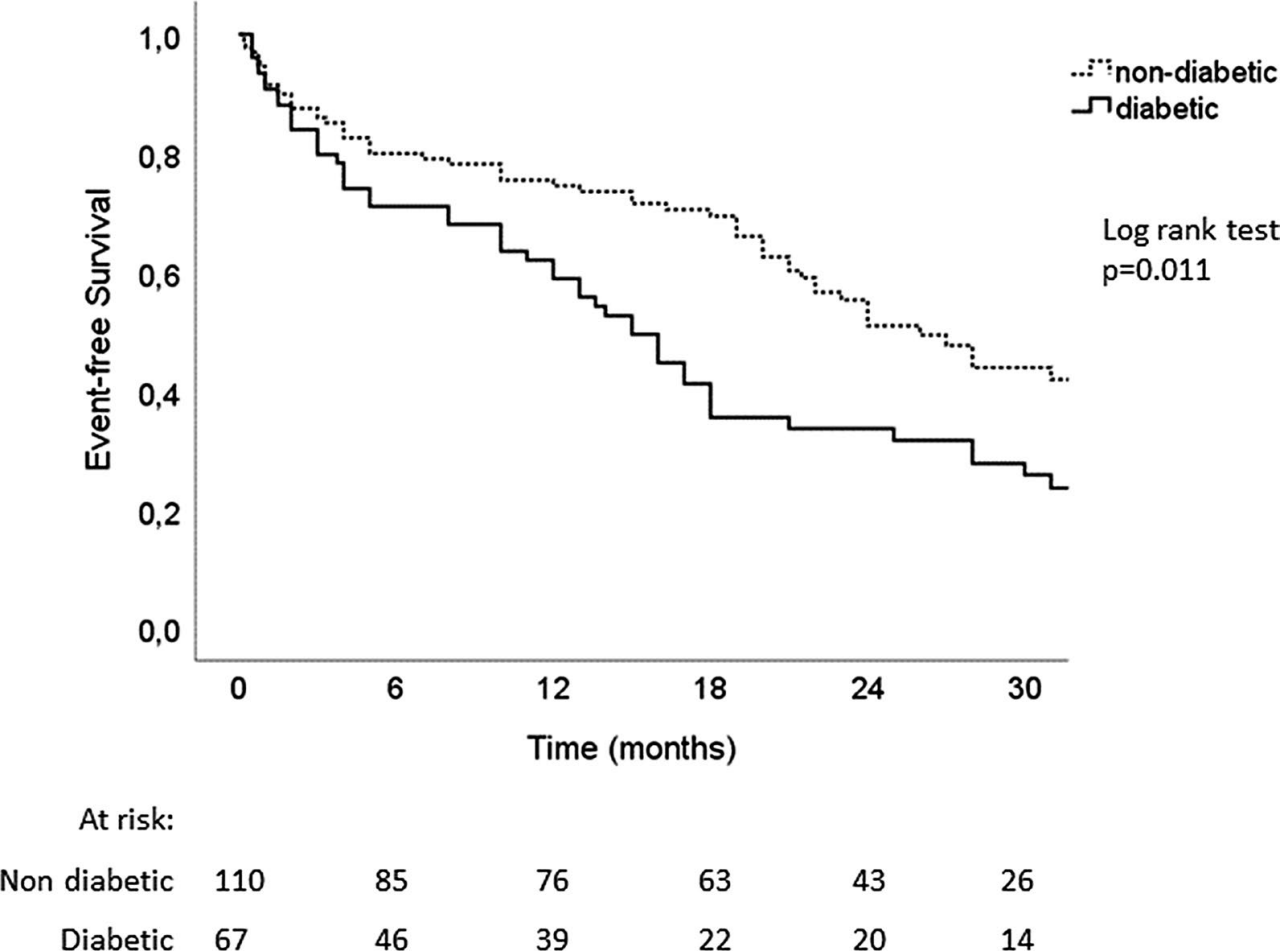
Kaplan Meier curves of event-free survival in diabetic versus non-diabetic HFpEF patients. Adjustments were made for age, body mass index, NYHA functional class and glomerular filtration rate

The follow up was completed for 177 (97%) patients, including 67 diabetics (96%) over a mean duration of 30 ± 9 months. Over this period of time, 27/67 (40%) diabetic patients died, and 52/67 (78%) reached the combined outcome, versus 28/110 (25%) deaths and 59/110 (54%) combined outcome in the nondiabetic group. As such, T2D was associated with worse prognosis in univariate Cox regression (HR 1.65 [1.1–2.4], p = 0.009). Although it shortly missed statistical significance for mortality alone, the association between diabetes and single outcomes taken separately went in the same direction (for all-cause mortality HR 1.58 [0.9–2.7], p = 0.092 and for hospitalization HR 1.64 [1.1–2.5], p = 0.022). After adjustment for age, body mass index, NYHA functional class and glomerular filtration rate, diabetes remained a significant predictor of mortality and hospitalization for heart failure (HR: 1.72 [1.1–2.6], p = 0.011) as shown by the adjusted Kaplan Meier curves (Fig. 2).

### ***Characteristics and outcome of diabetic HFpEF patients according to glycemic control (Table ******2******)***

**Table 2:** Clinical characteristics of diabetic HFpEF patients according to glycemic control

	Diabetic N = 65	HbA1C < 7% N = 32	HbA1C > 7% N = 33	p-value
Age (years)	76 ± 9	76 ± 8	75 ± 10	0.79
Duration of diabetes (years)	19.3 ± 8	19.2 ± 9	19.4 ± 8	0.96
Female (n, %)	42 (60%)	20 (62%)	20 (61%)	0.88
Body mass index (kg/m^2^)	31 ± 6	29 ± 6	32 ± 7	0.048
NYHA III–IV (n, %)	27 (39%)	14 (44%)	12 (36%)	0.54
Hospitalized for HF at inclusion (n,%)	41 (63%)	21 (66%)	20 (61%)	0.55
Atrial fibrillation (n, %)	37 (53%)	18 (56%)	16 (48%)	0.53
Ischemic cardiomyopathy (n, %)	33 (47%)	13 (41%)	18 (55%)	0.26
Smoking (n, %)	27 (39%)	10 (31%)	14 (42%)	0.35
Hypertension (n, %)	67 (97%)	32 (100%)	31 (94%)	0.49
Hypercholesterolemia (n, %)	49 (70%)	22 (69%)	24 (73%)	0.72
Sleep apneas (n, %)	12 (18%)	4 (13%)	8 (27%)	0.16
COPD (n, %)	7 (10%)	3 (9%)	3 (9%)	0.97
Biology				
HbA1C (%)	7.1 [6.1–7.8]	6.1 [5.8–6.5]	7.7 [7.2–8.4]	< 0.001 by design
eGFR (ml/min/1.73m^2^)	50 ± 24	49 ± 27	48 ± 18	0.78
Hemoglobin (g/dL)	11 ± 2	11 ± 2	12 ± 2	0.046
NT-proBNP (pg/mL)	1745 [955–3710]	2373 [1148–5264]	1464 [506–3696]	0.086
Antidiabetic treatment				
Insulin (n, %)	32 (46%)	11 (34%)	21 (64%)	0.018
Metformin (n, %)	31 (44%)	15 (47%)	13 (39%)	0.54
Sulfonylureas (n, %)	16 (23%)	9 (28%)	5 (15%)	0.20
Gliptins (n, %)	8 (11%)	4 (13%)	4 (12%)	0.96

Continuous variables are expressed as mean ± 1 standard deviation (SD) and categorical variables as count and proportion. p-values are derived from independent sample t-test or Chi square test when appropriate

HbA1C: glycated hemoglobin; NYHA: New York Heart Association; COPD: chronic obstructive pulmonary disease; GFR: glomerular filtration rate estimated by CKD-epi

**Fig. 3:**
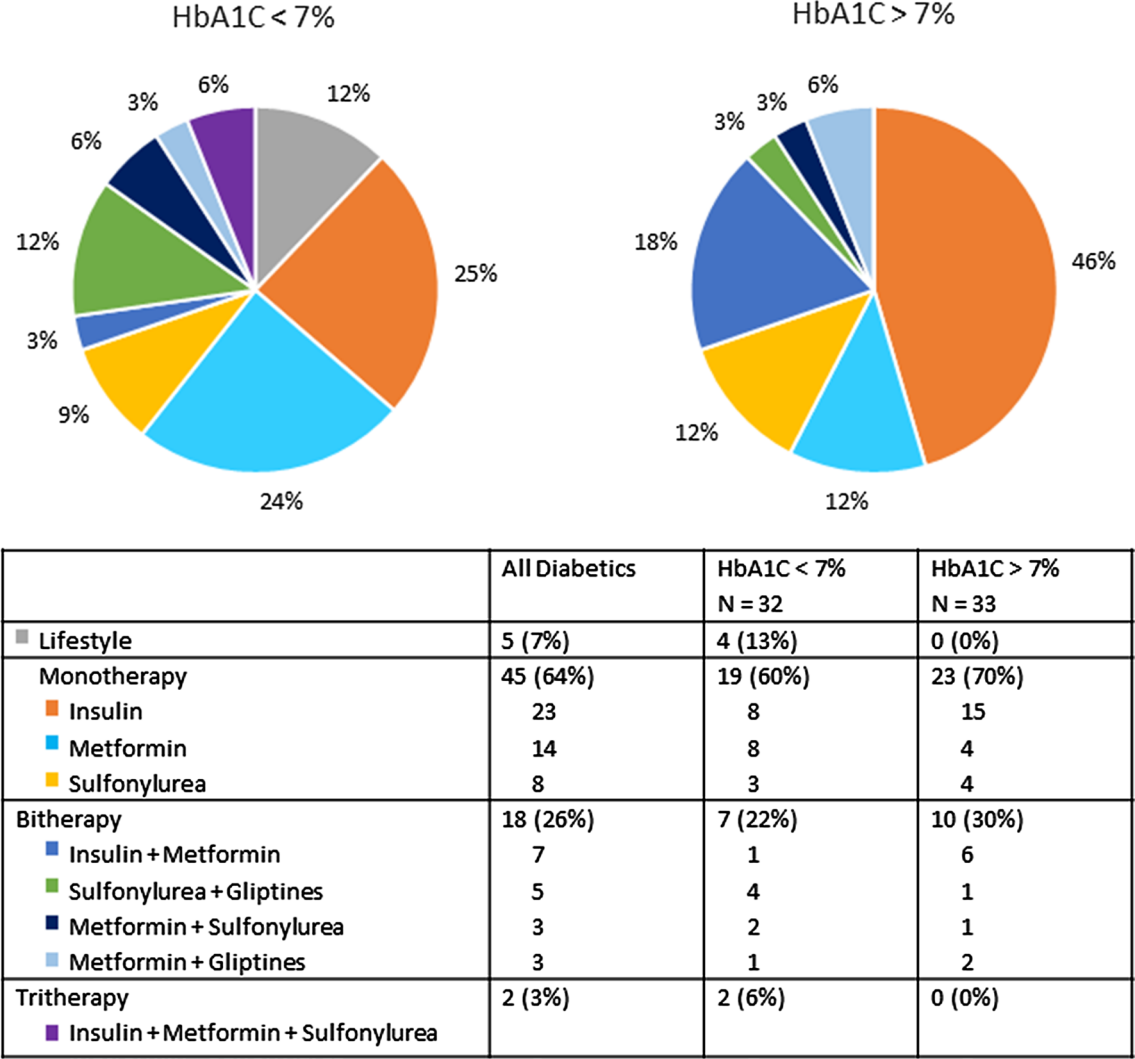
Hypoglycemic treatment of diabetic patients

Overall, the diabetic patients in our population had well controlled diabetes with median HbA1C of 7.1 [6.1–7.8] %. Almost half (32/65, 49%) were treated with insulin, alone or in combination with Metformin. Details of hypoglycemic treatments can be found in Fig. 3. Note that no patient was taking sodium-glucose cotransporter-2 inhibitors (SGLT-2i) as, in Belgium, they were reimbursed according to strict criteria at the time of inclusion. The subgroup of diabetic patients were compared among each other according to glycemic control (HbA1C < 7% versus > 7%, Table 2). Patients with HbA1C < 7% were leaner, with a mean BMI of 29 ± 6 versus 32 ± 7 kg/m^2^ (p = 0.048). They had slightly lower hemoglobin levels and showed a tendency, although not statistically significant, toward higher NT-proBNP levels. The two groups were homogenous regarding age, sex and comorbidities, and had similar renal functions. Patients with HbA1C > 7% were more often treated with insulin.

**Fig. 4:**
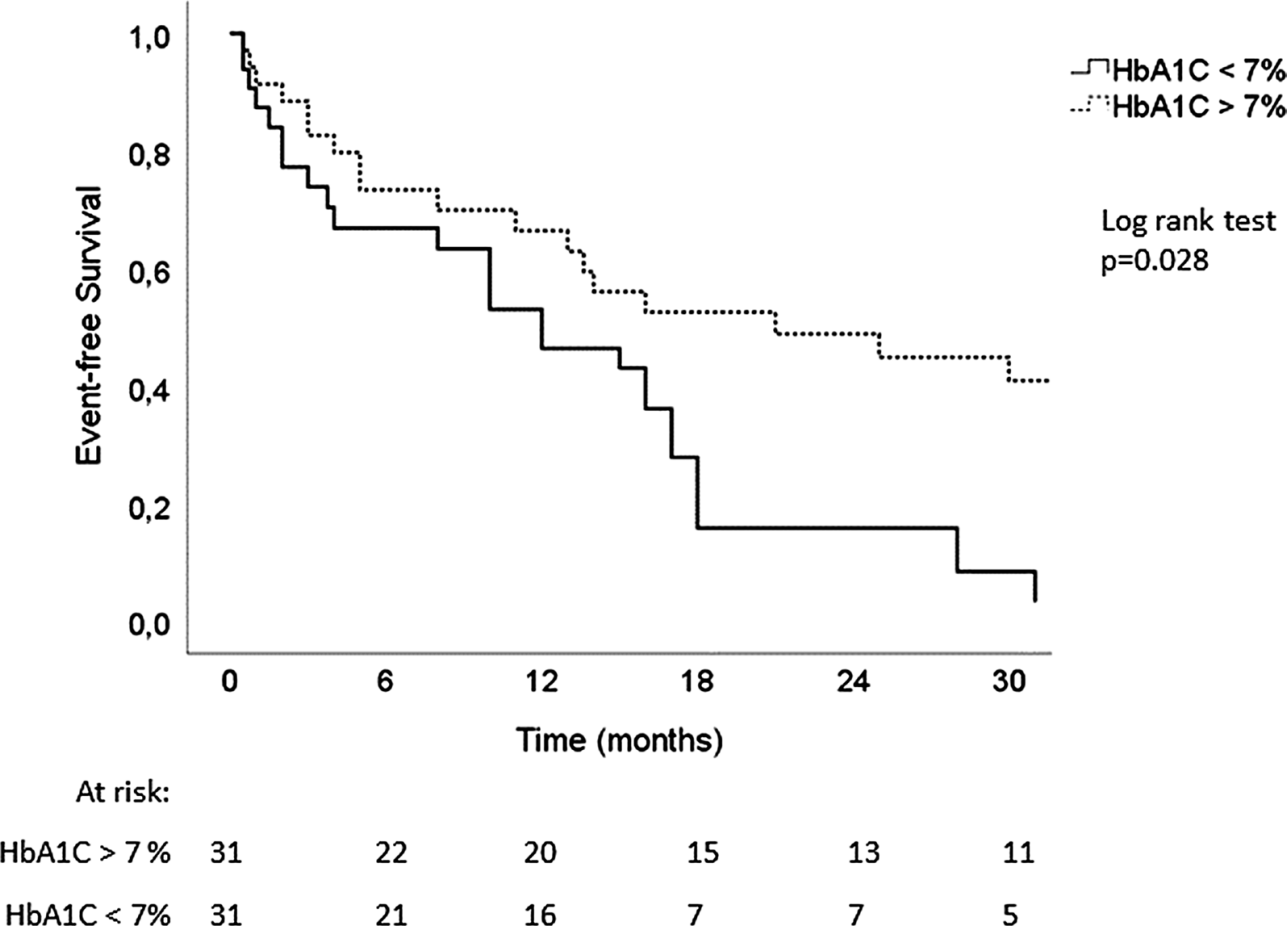
Kaplan Meier curves of event-free survival in diabetic HFpEF patients according to HbA1C levels. Adjustments were made for age, body mass index, hemoglobin and NTproBNP levels

Out of 65 diabetic patients with HbA1C data, 62 (95%) completed the follow up (Additional file 1). In 2 years, 15/31 (48%) diabetic patients with HbA1C < 7% died and 28/31 (90%) reached the combined outcome, versus 10/31 (32%) deaths in patients with HbA1C > 7% and 20/31 (65%) combined outcome. Lower levels of glycated hemoglobin were associated with worse prognosis (HR 2.07 [1.1–3.8], p = 0.016). Although it shortly missed statistical significance for hospitalization alone, the association between glycemic control and single outcomes taken separately went in the same direction (for all-cause mortality HR 2.36 [1.1–5.5], p = 0.047 and for hospitalization HR 1.86 [0.96–3.6], p = 0.064). After adjustment for age, body mass index, hemoglobin levels and NT-proBNP levels, HbA1C < 7% remained a significant predictor of mortality and hospitalization for heart failure (HR: 2.07 [1.1–4.0], p = 0.028). This can be seen in Fig. 4, showing the adjusted Kaplan Meier curves of event-free survival among diabetic HFpEF patients according to HbA1C levels.

## Discussion

The main findings of this study can be summarized as follows: 1. Diabetic patients with HFpEF show specific characteristics, including higher body mass index, lower prevalence of atrial fibrillation, lower hemoglobin levels and worse renal function. No echocardiographic difference could be detected, but CMR showed a trend towards higher LV mass and more myocardial fibrosis (ECV > 33%). 2. Diabetes is associated with an excess of adverse events (hospitalization for HF and mortality) in HFpEF. 3. Lower levels of HbA1C levels are associated with worse prognosis in diabetic patients with HFpEF.

### Characteristics and outcome of diabetic versus nondiabetic HFpEF patients

Regarding clinical characteristics, HFpEF patients with diabetes were younger and more obese than nondiabetic patients. This is consistent with sub studies from large clinical trials [6–8]. A large study examining age-related characteristics in HFpEF also observed that younger patients were more than twice as likely to be obese, and that the prevalence of diabetes ranged from 37% in the younger group versus 18% in the oldest group [19]. Although the reason for this difference is not completely elucidated, it might reflect that different pathophysiological pathways can lead to the development of HFpEF. The combination of diabetes and obesity, both conditions associated with a release of proinflammatory cytokines and decreased nitric oxide availability, could lead to the development of HFpEF at a younger age through myocardial remodelling and fibrosis [20]. Supporting this, diabetic patients also exhibited a trend towards higher LV masses and higher levels of myocardial fibrosis than their nondiabetic counterparts, consistently with previous studies [7, 21, 22]. This can contribute to the worse prognosis conferred by diabetes, as we previously showed that extracellular matrix expansion (higher ECV by CMR) was associated with adverse events in HFpEF [17].

Atrial fibrillation, on the other hand, was more prevalent in the nondiabetic group. This is consistent with previously published literature [23–25]. AF and HFpEF often coexist and it is still unclear whether one affection leads sequentially to the other. More likely, the two disorders share a common mechanistic substrate, which causes AF and HFpEF [26, 27] and develop in parallel. A recent meta-analysis underlined that AF was associated with poor prognosis in HFpEF, although it is unclear whether AF is only a marker of more severe heart failure, or a cause of mortality in itself [28]. Atrial fibrillation is also an age-related marker, hence, it is not surprising that the prevalence of AF is higher in the older nondiabetic group. Studies have also suggested differences in cardiac remodelling, with diabetic patients showing smaller LA volumes, that might contribute to this phenomenon [23]. However, the presence of AF was retrieved from medical files, patients interrogation, and a standard electrocardiogram at inclusion, but no long term rhythm monitoring was performed. As such, the prevalence of AF and other arrhythmias could have been underestimated in both groups.

The event rate in our study was high compared to clinical trials (16.1 / 100 persons-year overall mortality in the diabetic group versus 6.8–8.8 in pooled data from I-Preserve, Charm-Preserved and TOPCAT [23]), but similar to a large community based study (15.2/100 persons-year [29]). Compared to clinical trials, our population is almost 10 years older (76 vs 69 years) had higher NT-proBNP levels (1745 vs 430–581 pg/mL), lower hemoglobin (11 vs 12.9–13.5 g/dL) and worse renal function (50 vs 62.7–71.4 mL/min/1.73 m^2^), all parameters associated with adverse events. The association between diabetic status and prognosis (hospitalization for HF and mortality) is consistent with the existing literature [6, 7, 9, 10, 23]. There are numerous pathophysiologic processes in diabetes that are thought to alter the myocardium resulting in less effective relaxation and contraction, including oxidative stress, inflammation and disorders in calcium transport, as well as alterations in substrate metabolism, and mitochondrial dysfunction [30, 31]. Furthermore, extra-cardiac effects of diabetes such as decreased arterial compliance, renal angiopathy, and autonomic dysfunction can also accelerate the progression of HFpEF [31]. In particular, hyperglycemia causes up-regulation of the sodium-glucose cotransporter-2 (SGLT-2) leading to increased proximal renal sodium absorption, volume expansion, and decreased responsiveness to diuretics [20, 32, 33]. A better understanding of the interplay between diabetes and HF is crucial for the development of new therapies. This has recently been emphasized by the promising results of studies using SGLT-2 inhibitors in diabetic patients with HF [34, 35]. The results of ongoing randomized controlled trials using SGLT-2i in HFpEF [4, 20] are eagerly awaited. Nevertheless, a retrospective study showed less impressive effects of SGLT2i on cardiac remodeling in HFpEF compared to HFrEF, tempering enthusiasm for this class of treatment [35].

### Characteristics and outcome of diabetic HFpEF patients according to glycemic control.

While the presence of diabetes conferred a worse prognosis to our HFpEF patients, tight glycemic control did not seem to reverse this association. On the contrary, patients with best controlled diabetes (HbA1C < 7%) were more at risk for adverse events (hospitalization for heart failure and all-cause mortality). Previous studies [11, 12, 36, 37] showed a U-shaped association between HbA1C and prognosis in heart failure patients, with the lowest risk in the group of patients with HbA1C between 6.5 and 7.5%. However, those studies were either conducted among patients with HFrEF alone, or did not make a distinction between patients according to ejection fraction, while the interplay between diabetes and outcome seems to differ in those populations. In the CHARM trial, the relative risk conferred by diabetes was significantly greater in patients with preserved ejection fraction (EF) than in those with low EF [8] and a recent study highlighted that the presence of T2D was associated with a reduction of exercise capacity (lower peak VO2) in the LVEF < 40% and LVEF 40–49%, but not in the LVEF > 50% subgroup [38].

Data about glycemic control and outcome in HFpEF are scarce. A study by Gu et al. [13] did not find baseline HbA1C to be an independent predictor of outcome, but they analyzed it in the overall population of HF with T2D, and not only in HFpEF. Glycemic variability, however, was associated with outcome in the HFpEF subgroup [13] and was associated with signs of diastolic dysfunction in patients without HF [14]. Finally, the GAMIC cohort, a large population-based propensity-matched study of patients with HF [29] observed an increased mortality and morbidity (hospitalizations and visits) in patients who developed diabetes, particularly in those with a mean HbA1c higher than 7.0%.

How can we explain that, in our population, patients with higher HbA1C levels seem “protected” and suffer from less adverse events, while recent research emphasized the direct role of glucotoxicity on cardiomyocytes in the development of diabetic cardiomyopathy [31, 39, 40]? Firstly, glucotoxicity plays a part in the pathophysiology of the disease but its role in the evolution of symptoms and outcomes is yet unknown. Heart failure in diabetic patients occurs in a broad context of metabolic disorders including lipotoxicity, glucotoxicity and insulin resistance and resulting in impaired mitochondrial oxidative capacity and increased reactive oxygen species (ROS) production and surely, hyperglycemia is not the only mechanism involved. This is supported by the fact that, before the SGLT-2 inhibitor era, no study could demonstrate a favorable effect of glucose lowering therapies on events related to heart failure [41]. Conversely, some glucose-lowering therapies, including peroxisome proliferator-activated receptor (PPAR) agonists even increased the risk of heart failure in individuals with type 2 diabetes. Note that those drugs were seldom taken by patients in our cohort (Fig. 3) and cannot solely be responsible for the difference in event-free survival.

For years, it has been assumed that insulin resistance observed in diseases characterized by nutrient excess (ie T2D and obesity), was fundamental to the pathogenesis of these diseases. As stated above, insulin resistance into the heart has been considered to favor myocardial contractile dysfunction and to be involved in the pathophysiology of diabetic cardiomyopathy. However, an alternative view, which recently gained researchers’ interest, is that adaptations occurring in metabolic diseases can be viewed as protective in nature, and that insulin resistance could act as a defense mechanism to prevent or delay pathological intracellular substrate accumulation when substrate uptake exceeds energy demand [42–45]. Fundamental to this hypothesis is that, although these metabolic alterations are deleterious in the long term for complications associated with obesity and diabetes, they provide immediate protection against cell death in response to excess nutrients. Supporting this, it has been shown that cardiac contractile function was preserved, or even improved, in hearts subjected to metabolic and haemodynamic stress when myocardial insulin resistance was induced in response to elevated glucose levels or upon high-fat diet [46, 47]. Conversely, excessive insulin signaling exacerbates systolic dysfunction when the heart is subjected to pressure overload [48]. In light of this, the discrepancy between our study and the results of the GAMIC cohort [29] might be explained by the difference in disease duration. The GAMIC cohort excluded patients with a previous diagnosis of diabetes, while the mean duration of diabetes in our population was 19 ± 8 years. Possibly our results do not apply to new onset diabetes, as the adaptation to excess nutrients have not yet taken place.

In this context of old patients with long standing diabetes, the utility of therapeutically targeting glycemia, particularly through insulin sensitization, is questionable as it may result in exposure of cells and tissues to additional nutrients that will further challenge their survival. This could explain why PPAR agonists, important insulin sensitizers favouring nutrient uptake and storage, have been associated with adverse cardiovascular outcomes in T2D patients. On the other hand, treatment reducing nutrient overload might be beneficial and should be preferred. Metformin, for example, which has shown beneficial effect on mortality in HF patients [49], although often referred to as insulin sensitizer, has its main glucose-lowering effect via reducing hepatic glucose production. Similarly, SGLT-2 inhibitors lower blood glucose by promoting glycosuria.

This cardioprotective effect of insulin resistance could be involved in the better prognosis observed in heart failure patients with higher BMIs, referred to as the “obesity paradox” [15, 50]. Although we did not measure insulin resistance per se, we can hypothesize that the group with HbA1C > 7% is more insulin resistant as they are more obese and show higher glycemia levels though intensively treated.

Furthermore, hyperglycemia was shown to be involved in irreversible epigenetic changes, known as “glycemic memory”, and HbA1c at time of the study cannot reflect the whole history of diabetes [51, 52]*.* Similarly, intermittent hyperglycemia, rather than chronic elevation of blood glucose, with a lesser repercussion on HbA1C levels, exacerbates the production of reactive oxygen species, impairs endothelial function and induces cytokines release and contributes to pejorative evolution [53]. Finally, hypoglycemia could also be involved in the progression of cardiovascular diseases and mortality through sympatho-adrenal response [54].

In short, together with existing literature, this study underlines that other mechanisms besides glucotoxicity must be involved in the development and worsening of heart failure in diabetic patients, and that the effect of intensive glycemic control on cardiovascular associated morbidity is not fully understood.

Current guidelines recommend that the appropriate target for HbA1C should be individualized based on overall health and life expectancy. As such it is generally accepted that the glycemic goal should be somewhat higher (HbA1C ≤ 8%) in frail older adults with medical and functional comorbidities [55, 56]. Patients with HFpEF generally match this description (mean age of 78 years and high comorbidity burden in our population). However, these recommendations are based on consensus and there are virtually no trials that have examined glycemic control and complications focusing on the older patients, and even less on older patients with HFpEF. Hence, an important issue that is still unsolved is the optimal target level of HbA1c in that population. Given published data, glycemic variability should be avoided once the optimal target is reached [13]. Our study is a retrospective analysis of a relatively small population and does not allow answering this question. Furthermore, very few patients in our population had severely uncontrolled diabetes. However, this study generates the hypothesis that low levels of HbA1C are associated with more adverse events, and that physicians should not be too stringent about glycemic control in HFpEF patients with long standing diabetes. In addition, it underlines the need for future studies: fundamental studies to unravel the interaction between diabetes, insulin resistance and heart failure, and clinical studies designed to determine the optimal HbA1C target in HFpEF.

## Limitation

This study was conducted in a single center with a relatively small number of patients. Although data were collected prospectively, the association between HbA1C and mortality were derived from retrospective analyses. As such, this observation is subject to collider stratification bias and our data do not allow generalizing this finding beyond HFpEF patients. The diagnosis of diabetes was reported by investigators and did not require systematic documentation using standardized diagnostic criteria. Its prevalence is, therefore, likely to have been underestimated. Also, unmeasured confounders, such as biomarkers of nutritional status, invasive hemodynamics, and duration of heart disease, that may have improved risk adjustment, were unavailable.

## Conclusion

Together with previous data, this study suggests a potential differentiation of HFpEF phenotypes, with young obese and diabetic HFpEF on one hand, versus elderly HFpEF with atrial fibrillation on the other. This might reflect distinct pathophysiological pathways that perhaps should be targeted more specifically in future clinical trials*.* Furthermore, these results strengthen evidence on the prognostic significance of diabetes in HFpEF. It underlines that patients with HFpEF and diabetes are at high risk of hospitalization for HF and should benefit of closer monitoring and intensive treatment of comorbidities and congestion. Finally, it shows that a stringent glycemic control has a negative impact on prognosis. This opens the way for future research to better understand the interplay between diabetes and heart failure, and to determine an optimal HbA1C target in this specific population.

## Supplementary Information

**Additional file 1: Figure S1.** Flowchart of the study population. In blue, patients included in descriptive statistics (Tables 1 and 2). In red, patients with complete follow up, used for survival analysis. HbA1C: glycated hemoglobin.

## Data Availability

The datasets used and analyzed during the current study are available from the corresponding author on reasonable request.

## Abbreviations

AF: Atrial fibrillation
BMI: Body mass index
CMR: Cardiac magnetic resonance
ECV: Extracellular volume
EF: Ejection fraction
eGFR: Estimated glomerular filtration rate
HbA1C: Glycated hemoglobin
HFpEF: Heart failure with preserved ejection fraction
LA: Left atrium
LGE: Late gadolinium enhancement
LV: Left ventricle
NYHA: New York Heart Association
PPAR: Peroxisome proliferator-activated receptor
ROS: Reactive oxygen species
SD: Standard deviation
SGLT-2: Sodium-glucose cotransporter-2
T2D: Type 2 diabetes
TTE: Transthoracic echocardiography
