# Supplementary figures and images for: Diabetic phenotype and prognosis of patients with heart failure and preserved ejection fraction in a real life cohort
Source: Cardiovasc Diabetol. 2021 Feb 19;20:48. doi: 10.1186/s12933-021-01242-5 (PMC7893869; doi:10.1186/s12933-021-01242-5)

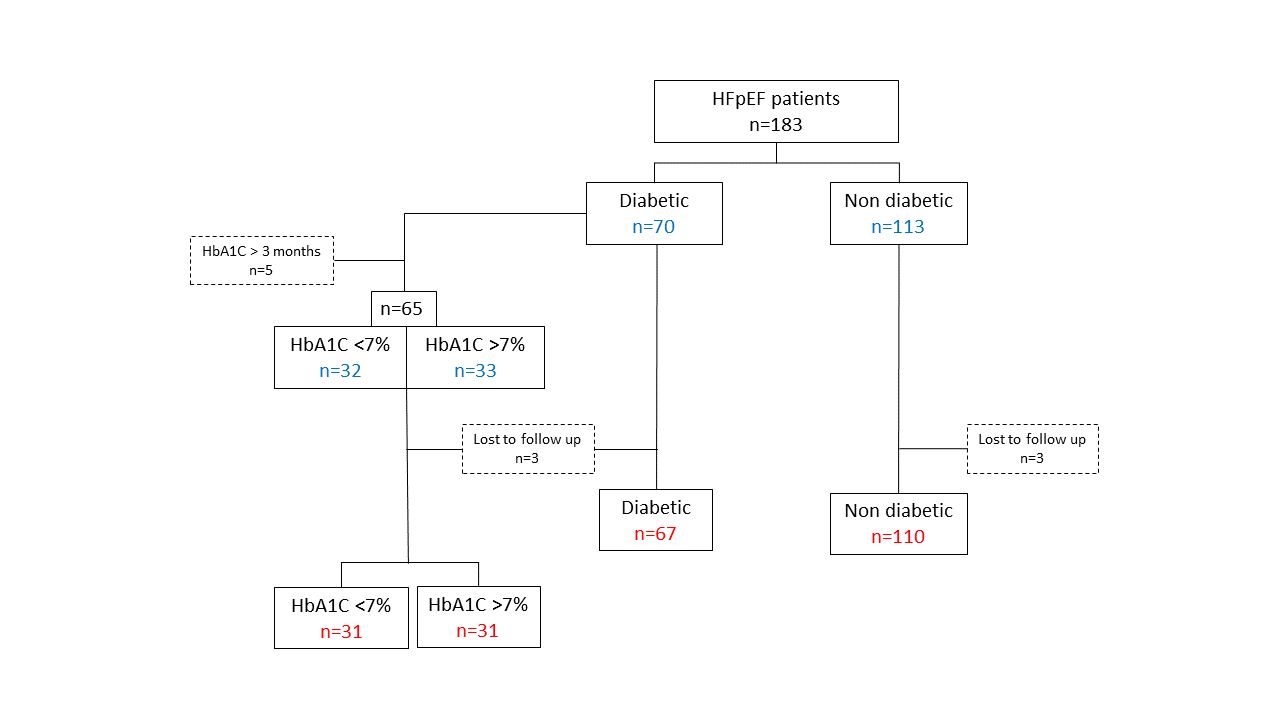

Supplement: Supplementary file 1 — Additional file 1: Figure S1. Flowchart of the study population. In blue, patients included in descriptive statistics (Tables 1 and 2). In red, patients with complete follow up, used for survival analysis. HbA1C: glycated hemoglobin. [file 12933_2021_1242_MOESM1_ESM.png]
